# The microbiota-gut-brain-epigenome axis as a novel therapeutic target for decoding postpartum depression

**DOI:** 10.3389/fmed.2026.1778348

**Published:** 2026-03-20

**Authors:** Boyuan Zheng, Xiaoming Shen, Ning Han, Xiao Guo, Shanrong Wan

**Affiliations:** 1 Henan University of Chinese Medicine, Zhengzhou, China; 2 Department of Obstetrics and Gynecology, The First Affiliated Hospital of Henan University of Traditional Chinese Medicine, Zhengzhou, China; 3 Department of Encephalopathy, The First Affiliated Hospital of Henan University of Traditional Chinese Medicine, Zhengzhou, China; 4 Department of Obstetrics and Gynecology, The Third Affiliated Hospital of Zhengzhou University, Zhengzhou, China

**Keywords:** choline metabolism, DNA methylation, epigenetics, genetic susceptibility, gut microbiota, microbiota-gut-brain axis, neurotransmitters, postpartum depression

## Abstract

**Background:**

Postpartum depression (PPD) is a psychological disorder affecting approximately 10–15% of women following childbirth, with significant implications for maternal and infant well-being. While hormonal fluctuations and psychosocial factors have long been considered primary contributors, recent reports demonstrated that gut microbiome is implicated in modulating maternal mood and behavior. The bidirectional communication between the gut and brain, mediated by microbiota-gut-brain axis, along with genetic and epigenetic modifications, has gained increasing attention as a potential mechanistic pathway in PPD. However, the precise genetic and epigenetic underpinnings of this interaction remain to be elucidated.

**Objective:**

This review aims to explore the genetic and epigenetic landscape of postpartum depression, with a significant focus pertinent to gut microbiota role in shaping neurobiological outcomes. By integrating recent findings from genomic, epigenomic, and microbiome research, we seek to elucidate novel mechanistic insights and potential therapeutic avenues.

**Methods:**

A comprehensive literature search was conducted using public databases, including PubMed, Google Scholar, and NCBI, to identify relevant studies on PPD, gut microbiota, genetics, and epigenetics.

**Results:**

Gut microbiota and neuroimmune modulation: peripartum changes in gut microbiota composition have been linked to immune dysregulation, inflammation, and neurotransmitter imbalances, all of which are implicated in PPD pathophysiology. Genetics and epigenetics of PPD: Genome-wide association studies (GWAS) revealed a profound genetic risk loci associated with PPD. Additionally, DNA methylation, histone modifications, and non-coding RNAs have profound functional implications in gene expression regulation, influencing PPD susceptibility. Epigenetic influence of the gut microbiome: The gut microbiome affects epigenetic modifications, such as DNA methylation and histone acetylation, which may lead to fetal programming and maternal mental health disorders. Choline metabolism and maternal mental health: Choline, an essential nutrient involved in epigenetic regulation, influences gut microbiota composition and brain function. Dysregulation in choline metabolism is associated with higher risk of PPD. Clinical and therapeutic implications: Understanding the genetic and epigenetic mechanisms underlying PPD offers new avenues for personalized therapeutic interventions, including probiotic and prebiotic strategies, microbiome-based treatments, and targeted epigenetic therapies.

**Conclusion:**

The interplay between genetics, epigenetics, and gut microbiota represents a novel and promising area of research in understanding postpartum depression. The microbiota-gut-brain axis serves as a crucial mediator in this relationship, influencing neuroimmune regulation, neurotransmitter synthesis, and epigenetic modifications. Future studies should focus on integrating multi-omics approaches to unravel the molecular complexity of PPD and develop targeted interventions aimed at restoring microbiome and epigenetic homeostasis.

## Graphical abstract

**Figure:**
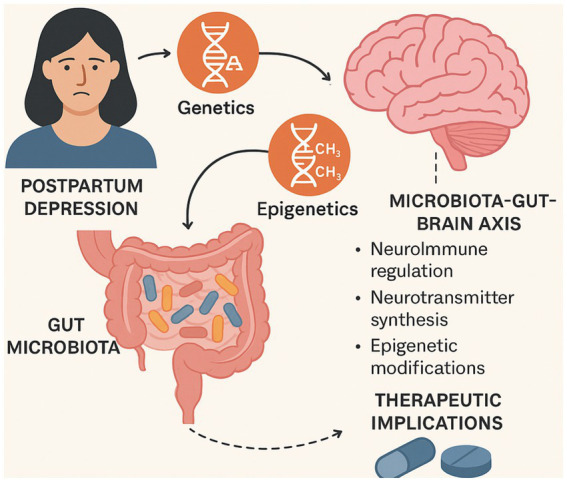
The image highlights emerging therapeutic implications, including microbiome-targeted and epigenetic-based interventions, positioning this integrative axis as a novel target in PPD research and management.

## Highlights

- Genetic and epigenetic factors: Examines how genetic predisposition and epigenetic modifications contribute to PPD pathophysiology.
- Gut microbiota and mental health: Explores how gut microbiota dysbiosis influences neuroimmune signaling and neurotransmitter synthesis in PPD.
- Choline metabolism and fetal programming: Highlights the role of choline metabolism in maternal mental health and its potential epigenetic effects on offspring.
- Therapeutic implications: Discusses microbiome-based and epigenetic interventions for managing and preventing PPD.
- Multi-Omics approach: Emphasizes the need for integrative genomics, epigenomics, and microbiome studies to uncover novel therapeutic targets.

## Introduction

Postpartum depression (PPD) is a significant mood disorder affecting 10–15% of postpartum women and PPD characterized by symptoms such as sadness, irritability, and feelings of hopelessness (1–3). This condition not only impacts the mother’s well-being but also has profound effects on family dynamics and child development (1–3). Recent research has highlighted the role of genetic and epigenetic factors in the etiology of PPD, which yet to be explored vividly using future studies (1–4). Genetic predispositions, including polymorphisms in genes related to neurotransmitter systems (1–4), hormonal pathways, and immune responses, have been proposed based on broader depression studies which may be associated with increased susceptibility to PPD. Epigenetic mechanisms, such as DNA methylation and histone modifications, can modulate gene expression without altering DNA sequence, potentially affecting mood regulation and stress responses. For instance, variations in the oxytocin receptor gene (OXTR) methylation have been linked to PPD, suggesting that epigenetic alterations may play a prominent function in the onset of this disorder (1–6).

Gut-brain axis refers to the complex bidirectional communication confined to human gut and CNS, encompassing neural, hormonal as well as immunological signaling pathways (1–4, 7). Gut microbiota is composed of microbial milieu, which organize this axis by influencing brain function as well as behavior. Alterations in gut microbiome composition is implicated in several neuropsychiatric disorders, such as depression and anxiety (1–4). Previous reports (8, 9) demonstrated that the gut microbiota may significantly modulate development of PPD. In addition, peripartum changes in the gut microbiota composition can affect maternal mood and behavior (1–4, 7). For example, disruptions in gut microbial balance have been associated with increased inflammation and altered neurotransmitter production, both of which are linked to depressive symptoms. Moreover, specific bacterial genera such as *Alphaproteobacteria, Roseburia, Alistipes onderdonkii, Bilophila wadsworthia* (10) have been found to correlate with mood regulation, indicating that the gut microbiota could be a potential target for therapeutic interventions in PPD (1–4, 7).

Enteric nervous system (ENS), an intricate network of neurons embedded within the gastrointestinal (GI) tract, functions as a key component of autonomic nervous system and can be considered as “second brain” (2, 11). Owing to its dense autonomic innervation and the presence of an extensive enteric nervous system with autonomous sensory, motor, and integrative capabilities, the gut functions as a “second brain,” exerting profound influence over immune regulation, neuroendocrine signaling, and brain function (2, 11). ENS regulates a diverse array of physiological processes, including motility, secretion, absorption, and immune responses, primarily through neurotransmitters generation and gut-derived hormones (11). In parallel, CNS (2, 11) modulates GI function by neural, endocrine, and immune signalings, collectively forming the bidirectional gut-brain axis. This complex communication system ensures homeostasis but also represents a double-edged sword, while a balanced gut microbiome promotes health, microbial dysbiosis, characterized by alterations in microbial composition, has been implicated in a spectrum of pathologies, such as inflammatory bowel disease, metabolic disorders, and neuropsychiatric conditions (2, 11).

Mood and anxiety disorders represent a significant global health burden, exhibiting a more than 50% raise for the past 30 years, thereby concluding the urgency of identifying novel therapeutic targets (2, 7, 12, 13). The gut microbiome has emerged as a critical modulator of mental health, influencing mood regulation through multiple mechanisms, including modulation of neurotransmitter synthesis (e.g., serotonin, gamma-aminobutyric acid), immune system regulation, and the hypothalamic–pituitary–adrenal (HPA) axis. Perturbations in microbial diversity and composition have been linked to heightened systemic inflammation, oxidative stress, and disrupted neurochemical balance, contributing to the pathogenesis of psychiatric disorders (2, 14). The interplay between genetic, epigenetic, and gut-brain axis factors presents a multifaceted framework for understanding PPD. Genetic predispositions may influence the composition and function of the gut microbiota, while epigenetic modifications can modulate the expression of genes involved in immune responses and neurotransmitter systems. This intricate network suggests that interventions targeting the gut microbiota, such as probiotics or dietary modifications, could potentially mitigate PPD symptoms by influencing both genetic and epigenetic pathways. Advancements in understanding the genetic and epigenetic underpinnings of PPD (2), alongside the pivotal role of the gut-brain axis, offer promising avenues for novel therapeutic strategies. Future research integrating these domains is essential to develop personalized interventions that can effectively address the complex etiology of PPD, ultimately improving outcomes for affected women and their families. In this review, we described the genetic and epigenetic landscape of postpartum depression, with a particular emphasis on the role of gut microbiota in influencing neurobiological outcomes (2). By synthesizing recent advancements in genomic, epigenomic, and microbiome research, this study aims to uncover novel mechanistic insights and identify potential therapeutic strategies.

## Methodology

### Study design

This work was conducted as a systematic narrative review without a comprehensive meta-analysis, with the objective of systematically identifying, synthesizing, and contextualizing the existing evidence on genetic, epigenetic, and gut microbiota-related mechanisms implicated in PPD. The review emphasizes qualitative evidence synthesis across heterogeneous study designs rather than quantitative effect estimation.

The review process followed PRISMA-informed reporting principles to enhance transparency in literature identification, screening, and selection. However, given the conceptual and mechanistic focus of the review, as well as the diversity of included study designs, a formal meta-analysis and quantitative risk-of-bias scoring were not undertaken. The primary aim was to integrate findings from genomics, epigenomics, and microbiome research to explore how these biological domains converge through the microbiota-gut-brain axis and contribute to maternal mental health, while identifying emerging mechanistic pathways and translational implications for PPD prevention and management.

### Search strategy

A systematic literature search was performed to identify relevant studies published between January 2010 and June 2025. The following electronic databases were searched: PubMed, Relmed, Embase, Scopus, and Web of science. Database-specific search strategies were developed using combinations of controlled vocabulary (where applicable) and free-text terms, linked by Boolean operators to maximize sensitivity and specificity.

The core search terms included combinations of the following keywords: “postpartum depression” OR “maternal depression,” AND “gut microbiota” OR “microbiome” AND “epigenetics” OR “DNA methylation” OR “histone modification” OR “non-coding RNA” AND “genetics” OR “GWAS” OR “gene expression” AND “microbiota-gut-brain axis” OR “neuroimmune modulation” AND “choline metabolism” OR “neurotransmitters.” To ensure comprehensive coverage, manual screening of reference lists from included articles and relevant reviews was also conducted to identify additional studies not captured in the electronic database searches.

### Eligibility criteria

Explicit inclusion and exclusion criteria were applied to ensure consistency and methodological rigor. Studies were eligible for inclusion if they met the following criteria: (1) published in peer-reviewed journals and written in English, (2) original research articles, systematic reviews, or meta-analyses, (3) investigated postpartum depression in relation to gut microbiota composition, genetic susceptibility, or epigenetic mechanisms, (4) included human studies and/or animal models with relevance to PPD pathophysiology, (5) employed mechanistic or multi-omics approaches, such as genome-wide association studies (GWAS), epigenome-wide association studies (EWAS), transcriptomic analyses, or high-throughput microbiome profiling.

Studies were excluded if they were editorials, commentaries, conference abstracts, or reports lacking sufficient methodological detail. Articles focusing exclusively on major depressive disorder without specific relevance to the postpartum period were also excluded. Additionally, studies with unclear outcomes, insufficient mechanistic insight, or limited scientific rigor were not considered.

### Data collection and extraction

Data extraction was performed independently by at least two reviewers to minimize selection bias and enhance accuracy. Discrepancies were resolved through discussion and consensus. For each included study, the following information was systematically extracted: author(s), year of publication, study design, sample characteristics (e.g., maternal age, postpartum duration), type(s) of omics data analyzed (genetic, epigenetic, microbiome-related), and principal findings related to PPD, including alterations in the microbiota-gut-brain axis, immune modulation, and neurobiological pathways. Reported or proposed therapeutic and translational implications were also documented where available.

### Study selection

**Figure 1:**
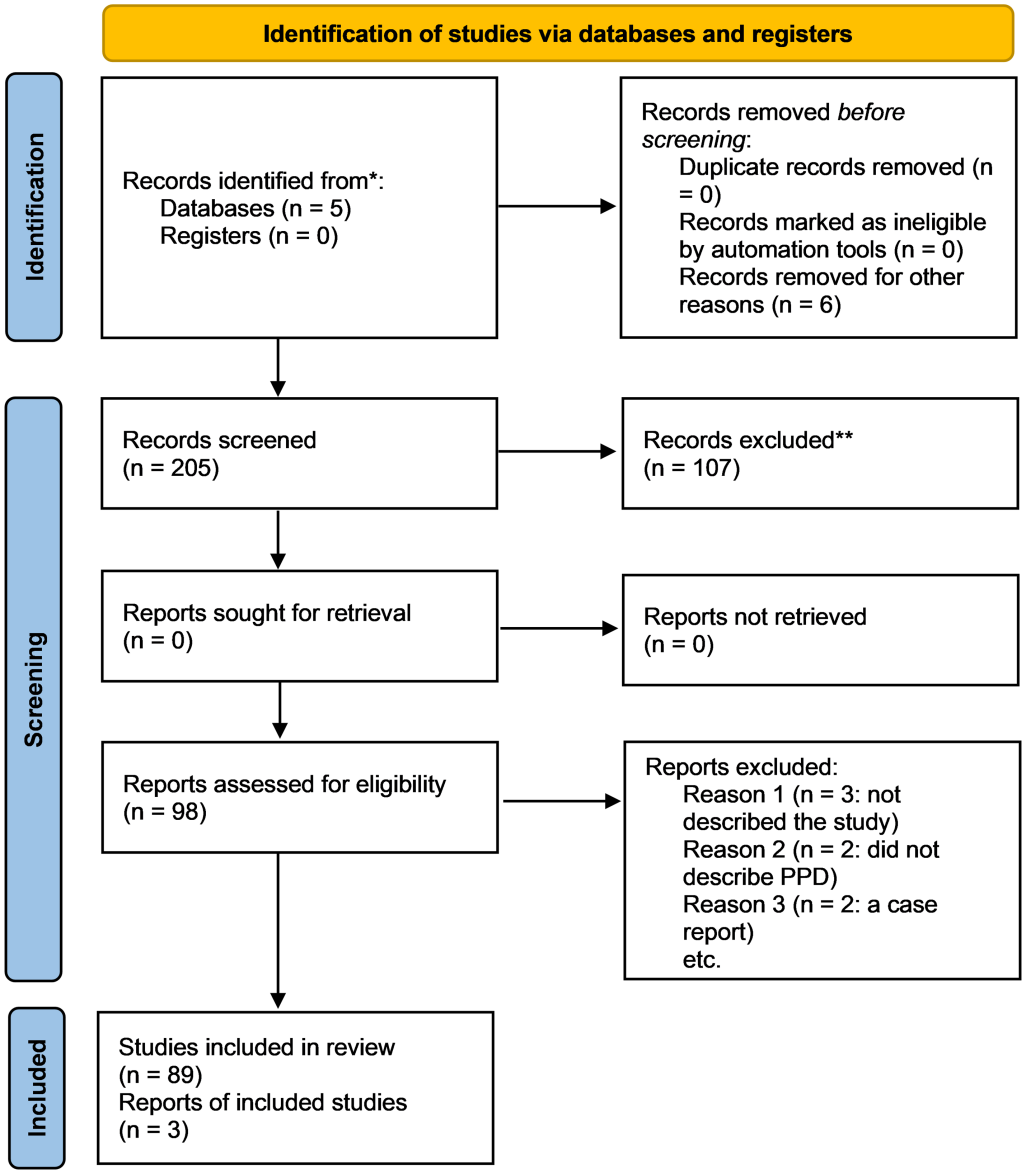
Schematic depiction of PRISMA flow chart as pertinent to this study.

The initial database search yielded 161 records. After removal of duplicate entries, unique records were screened based on titles and abstracts. During this stage, 63 records were excluded due to lack of relevance, topic mismatch, or methodological limitations. The remaining 98 full-text articles were assessed for eligibility. Following detailed evaluation, 7 full-text articles were excluded for reasons including insufficient mechanistic focus, inadequate data, or lack of relevance to the integrated gut microbiota-genetics-epigenetics framework in PPD. Ultimately, 91 studies were included in the final qualitative synthesis. All screening and selection steps were performed manually without the use of automation tools. A PRISMA-style flow diagram summarizing the study selection process is provided in Figure 1.

### Data synthesis

The extracted data were organized and synthesized thematically across the following domains: (1) gut microbiota alterations during the PPD; (2) genetic susceptibility and risk loci associated with PPD; (3) epigenetic mechanisms, including DNA methylation, histone modifications, and non-coding RNAs; (4) bidirectional interactions between gut microbiota and epigenetic regulation; (5) the role of choline metabolism and related neurobiological pathways in maternal mental health; and (6) emerging therapeutic strategies and translational perspectives relevant to PPD. This structured qualitative synthesis enabled integration of mechanistic insights across biological scales while maintaining transparency and reproducibility in the review process.

### Clinical manifestations of PPD, genetic and familial or societal influences on PPD

PPD is a major psychiatric disorder affecting approximately 10–15% of postpartum women, presenting with a constellation of symptoms including profound sadness, irritability, anhedonia, crying spells, and, in severe cases, hallucinations and suicidal ideation (2, 15, 16). According to the Diagnostic and Statistical Manual of Mental Disorders, Fifth Edition, Text Revision (DSM-5-TR), PPD is defined as a major depressive episode with onset occurring within 6 months following childbirth, although broader clinical definitions extend this timeframe to encompass up to 12 months postpartum (2, 15, 16). The pathogenesis of PPD is multifactorial, with both biological and psychosocial determinants playing a pivotal role. Established risk factors include perinatal anxiety, psychiatric history, socioeconomic adversities, hormonal fluctuations, sleep deprivation, and obstetric complications (2, 11, 14–18). Notably, PPD is not confined to mothers; up to 70% of pregnant women experience transient depressive episodes during gestation, and recent epidemiological studies indicate that fathers are also susceptible, with paternal PPD prevalence estimated at 8.4% (2, 11, 14–18).

While environmental and psychosocial factors contribute substantially to PPD risk, genetic predisposition and familial aggregation of the disorder suggest a heritable component. Women with a first-degree relative (e.g., mother or sister) affected by PPD exhibit an increased likelihood of developing the condition themselves (2, 19). Genetic studies have implicated polymorphisms in genes regulating the serotonergic system, the HPA axis, and neuroimmune pathways in susceptibility to PPD. Furthermore, epigenetic modifications, such as altered DNA methylation pertinent to genes involved in stress responses and neuroplasticity, have been proposed as mechanistic links between environmental stressors and PPD development (2, 19). The natural course of PPD is variable, with symptom severity ranging from mild to severe depressive episodes. While many cases resolve with appropriate treatment, longitudinal studies suggest that approximately 24% of affected women remain depressed 1 year postpartum, and 13% continue to experience depressive symptoms 2 years after delivery (20). Importantly, PPD must be differentiated from “postpartum blues,” a transient mood disturbance affecting up to 80% of new mothers, typically resolving within 2 weeks without medical intervention (21, 22). In contrast, postpartum psychosis represents a rarer but more severe psychiatric emergency, often requiring immediate hospitalization due to the heightened risk of self-harm and infanticide (21, 22).

Beyond its profound impact on maternal well-being, PPD imposes a significant socioeconomic and medical burden on families and healthcare systems. Affected mothers often struggle with impaired bonding and reduced responsiveness to their infants, which can disrupt early childhood development, leading to long-term consequences such as delayed language acquisition, cognitive deficits, and a higher sensitivity to psychiatric disorders in later life (23–25). Children of mothers with untreated PPD are more likely to exhibit behavioral problems, emotional dysregulation, and academic difficulties (26). The adverse effects of PPD extend to the entire family unit, exacerbating marital conflict and contributing to household instability. Alarmingly, suicide has been identified as the second leading cause of maternal mortality in the postpartum period, emphasizing the urgent need for early detection and intervention strategies to mitigate the impact of this disorder (2, 27).

### Implications of the gut microbiome in depression and PPD: microbial metabolites and immune modulation

Postpartum depression (PPD) is a common and debilitating mood disorder that emerges during the perinatal period and uniquely affects maternal well-being, mother–infant interactions, and long-term developmental outcomes for offspring (2, 28). Unlike major depressive disorder outside the reproductive context, PPD arises against a backdrop of profound hormonal, immune, metabolic, and psychosocial transitions that render the postpartum brain particularly vulnerable. Understanding the PPD-specific pathophysiology is therefore essential for developing targeted and effective therapeutic strategies (2, 28). Epidemiological data indicate that the clinical burden of mood disorders during the postpartum period is substantial and frequently managed with antidepressant pharmacotherapy (2, 29). Although these data reflect broader prescribing trends, they underscore the continued reliance on symptom-oriented treatments in perinatal mental health care. Importantly, conventional antidepressant approaches often fail to address the biological mechanisms uniquely relevant to PPD, including postpartum-specific neuroendocrine shifts, immune activation, and microbiota–brain interactions. This highlights a critical gap in current treatment paradigms and reinforces the need for mechanistically informed, PPD-focused research (2, 29).

Emerging evidence suggests a strong association between gut microbiota composition and depression. However, current research primarily establishes a correlation rather than a definitive causal link (2, 29). This presents a significant challenge in understanding the precise mechanisms through which the microbiome contributes to psychiatric disorders. Rather than solely examining microbial composition, recent studies highlight the importance of investigating the biochemical pathways and microbial metabolites that influence host physiology (2, 30). Gut microbiota serves as a critical mediator in the gut-brain axis, facilitating bidirectional communication between gut and CNS through biochemical signaling pathways (2). Key metabolites such as serotonin, kynurenine, tryptophan, and short-chain fatty acids (SCFAs) play significant functions in neurochemical regulation, influencing mood, cognitive function, and neuroinflammation (2). Dysbiosis-induced alterations in these metabolic pathways can contribute to the pathogenesis of depression by disrupting neurotransmitter homeostasis and immune system regulation (2).

Among the various microbial metabolites, SCFAs, including butyrate, propionate, and acetate, have gained considerable attention for their neuroprotective and anti-inflammatory properties. Mainly, butyrate is regarded as fundamental energy source for colonocytes, that promotes intestinal barrier integrity and exerting protective effects against colorectal cancer and inflammatory diseases by blocking HDACs (2, 31). While all SCFAs exhibit HDAC inhibition, butyrate selectively modulates key receptors include GPR41/FFAR3, GPR43/FFAR2, and hydroxycarboxylic acid receptor GPR109A/HCAR2, which are implicated in mitigating pro-inflammatory cytokines generation (2, 32). SCFAs also play a crucial role in psychiatric disorders, as lower levels of butyrate have been observed in patients with depression (2, 33). By influencing BDNF expression, SCFAs contribute to neuronal plasticity and cognitive function. Additionally, they regulate the permeability of the blood–brain barrier, modulate immune responses, and impact neuroinflammatory processes, all of which are critical in the pathophysiology of depression (2).

Beyond their direct influence on neurophysiology, SCFAs are integral to immune system regulation (2). For instance, propionate has been shown to suppress pro-inflammatory cytokines include IL-1β and IL-18 in lung macrophages, thereby modulating immune responses and protecting against tissue injury in murine models (2). Additionally, propionate induces a metabolic shift in macrophages from glycolysis to oxidative phosphorylation following lipopolysaccharide (LPS) exposure, further demonstrating its role in immune homeostasis (2, 34).

Dietary composition significantly impacts microbial metabolite production. High-fiber diets are metabolized by gut bacteria to yield SCFAs, which contribute to gut homeostasis and systemic health (2). However, excessive fiber intake may paradoxically reduce microbial diversity, potentially leading to dysbiosis and altered metabolic outputs. This highlights the delicate balance required in dietary interventions aimed at modulating gut microbiota for mental health benefits (2).

Gut microbiome plays an crucial role in neuropsychiatric health, influencing depression through intricate biochemical pathways and immune modulation (2). SCFAs, particularly butyrate and propionate, serve as key mediators in maintaining gut integrity, regulating inflammation, and modulating neurochemical balance (2). Future research should focus on elucidating causal relationships between microbial metabolites and depression while exploring microbiota-targeted therapies as potential adjunctive treatments for mental health disorders (2). A comprehensive understanding of these mechanisms could pave the way for innovative therapeutic strategies that extend beyond traditional antidepressants, offering more effective and personalized interventions for depression (2).

### Recent advances and challenges in PPD treatment and neurobiological implications

PPD and anxiety are significant public health concerns that have profound effects on both maternal well-being and infant development. Despite extensive research efforts, PPD treatment continues to face considerable challenges due to its complex pathophysiology, the heterogeneity of patient responses, and the interplay between genetic, neurobiological, and environmental cues. The recent studies discussed here provide novel insights into the neurobiological underpinnings of PPD, its impact on infant neurodevelopment, and emerging therapeutic interventions.

Recent functional MRI (fMRI) studies provide compelling neurobiological evidence linking maternal postpartum depression (PPD) to alterations in early infant brain development, particularly within circuits supporting social cognition and emotional regulation (35). In healthy mother-infant dyads, exposure to the maternal voice robustly activates dopamine- and oxytocin-enriched brain regions in the infant, including the anterior cingulate cortex, temporoparietal junction, and striatum areas critical for reward processing, social salience, and attachment formation (35). In contrast, infants born to mothers exhibiting elevated PPD and anxiety symptoms show significantly attenuated activation within these same regions (35). This blunted neural responsiveness suggests early disruption of social reward circuitry and impaired encoding of maternal cues, potentially reflecting reduced dopaminergic and oxytocinergic signaling during sensitive periods of neurodevelopment (35). Given the central role of these neurotransmitter systems in shaping synaptic plasticity and social learning, such alterations may predispose offspring to long-term socioemotional and cognitive vulnerabilities. Importantly, these neuroimaging findings align with broader evidence implicating PPD-associated dysregulation of maternal affect, stress physiology, and caregiving behaviors in shaping infant brain development (35). Together, they describe the critical importance of timely identification and intervention for maternal PPD during the postpartum period, not only to improve maternal mental health outcomes but also to safeguard the integrity of early social brain network development in offspring (35).

### Dopaminergic dysregulation in postpartum psychosis (PP) and relevance to PPD

Increasing attention has been directed toward the role of dopamine signaling in postpartum psychiatric disorders, particularly postpartum psychosis (PP) (35). Neuroimaging studies using neuromelanin-sensitive MRI have demonstrated elevated neuromelanin levels in the substantia nigra and ventral tegmental area in women with a history of PP, suggesting altered midbrain dopamine turnover (35). These abnormalities were associated with subclinical psychotic symptoms and reduced functional connectivity between the substantia nigra and subcortical regions including the thalamus and hippocampus. While PP and PPD are clinically distinct entities, they share overlapping vulnerability windows related to abrupt hormonal withdrawal and postpartum neuroplasticity. Thus, dopaminergic alterations identified in PP may provide indirect insight into postpartum-specific neural susceptibility, although their relevance to PPD requires direct empirical validation through longitudinal and disorder-specific studies.

### Glucocorticoid signaling and cortico-cortical pathway dysfunction in PPD

Adverse life experiences (ALEs) have been implicated in the development of PPD through sustained dysregulation of the hypothalamic–pituitary–adrenal (HPA) axis. Women with a history of mental illness exhibited prolonged elevations in cortisol levels postpartum, indicating impaired HPA axis regulation. In parallel, rodent models have demonstrated that exposure to adolescent social isolation leads to postpartum behavioral disturbances, reduced social novelty preference, and decreased activity in the anterior insula-prelimbic cortex (AI-PrL) pathway (36). Notably, pharmacological blockade of glucocorticoid receptors normalized these behavioral deficits, whereas selective serotonin reuptake inhibitors (SSRIs) and GABAergic modulators did not. These findings suggest that targeting glucocorticoid signaling within specific cortico-cortical circuits may represent a novel therapeutic avenue for PPD treatment, warranting further translational research to explore its clinical applicability (36).

### Advancements in PPD pharmacotherapy: role of zuranolone

Zuranolone, a neuroactive steroid and positive allosteric modulator of GABA-A receptors, has emerged as a promising candidate for PPD treatment. Recent clinical trials have demonstrated its efficacy in rapidly alleviating depressive symptoms within 2 weeks of administration, a stark contrast to conventional antidepressants that typically require weeks to months to exert full therapeutic effects. Its rapid onset of action may be particularly advantageous for postpartum mothers, where prompt symptom relief is crucial for maternal–infant bonding and caregiving. However, further research is needed to evaluate its long-term safety, efficacy across diverse patient populations, and potential neurodevelopmental effects on offspring (37).

The integration of neuroimaging, genetic, and pharmacological studies is essential for advancing our understanding of PPD pathophysiology and optimizing treatment strategies. Key areas for future research include: Longitudinal studies to determine whether early alterations in infant brain function persist into later childhood and adolescence. Identification of predictive biomarkers for PPD susceptibility and treatment response, potentially leveraging neuromelanin-sensitive MRI and HPA axis profiling. Investigation of combination therapies that target both dopaminergic and glucocorticoid systems to enhance therapeutic outcomes in treatment-resistant PPD cases. Examination of the impact of novel pharmacotherapies, such as zuranolone, on maternal cognition, infant development, and long-term psychiatric outcomes (37). In conclusion, recent advancements in PPD research have provided invaluable insights into its neurobiological basis and potential intervention strategies. The identification of neural markers associated with maternal and infant brain function offers a promising avenue for early detection and personalized treatment approaches. Addressing the multifaceted nature of PPD through interdisciplinary research and precision medicine holds great promise for improving maternal mental health and optimizing early-life neurodevelopmental outcomes for future generations. Future efforts should also explore the potential interplay between genetic susceptibility, immune system dysregulation, and environmental stressors to develop comprehensive, individualized treatment approaches.

### Mechanistic links between epigenetic regulation and PPD-specific neural circuits

Emerging evidence indicates that epigenetic mechanisms in PPD are not merely descriptive molecular correlates but actively modulate stress-, plasticity-, and bonding-related neural circuits that are uniquely vulnerable during the postpartum period (38, 39). These mechanisms converge on the hypothalamic–pituitary–adrenal (HPA) axis, neurotrophic signaling, and oxytocinergic pathways, which together shape maternal stress responsivity, affect regulation, and mother-infant bonding (38, 39).

Epigenetic regulation of HPA-axis genes in PPD: DNA methylation and chromatin remodeling of glucocorticoid signaling genes represent a central mechanistic pathway linking stress exposure to PPD vulnerability. Increased methylation of the NR3C1 promoter (40), encoding the glucocorticoid receptor, has been associated with impaired negative feedback regulation of the HPA axis, resulting in prolonged cortisol signaling and heightened stress sensitivity in postpartum women (38, 39). Longitudinal cohort studies demonstrate that elevated NR3C1 methylation during late pregnancy predicts higher postpartum depressive symptom severity, particularly among women exposed to antenatal psychosocial stress or inflammatory burden (38–40).

Similarly, epigenetic modification of FKBP5, a key co-chaperone regulating glucocorticoid receptor sensitivity, has been implicated in stress-related psychiatric disorders, including postpartum mood disturbances. Hypomethylation of FKBP5 intronic enhancer regions enhances glucocorticoid receptor resistance, thereby sustaining HPA-axis hyperactivation. Postpartum-relevant FKBP5 methylation patterns have been linked to exaggerated cortisol responses and impaired stress recovery, suggesting a mechanistic epigenetic bridge between perinatal stress exposure and maladaptive maternal affective states (38–42).

*Epigenetic control of BDNF and neuroplasticity*: Neuroplasticity within limbic and prefrontal circuits is essential for postpartum emotional adaptation, and epigenetic regulation of BDNF plays a pivotal role in this process. Reduced histone acetylation and increased DNA methylation at BDNF promoters have been consistently observed in depressive phenotypes, leading to diminished synaptic plasticity in the hippocampus and medial prefrontal cortex regions critically involved in mood regulation and maternal responsiveness (43–45). In perinatal and stress-based models, epigenetically mediated repression of BDNF via histone deacetylase (HDAC) recruitment results in impaired dendritic remodeling and affective dysregulation resembling postpartum depressive phenotypes (45). Importantly, postpartum-relevant environmental modifiers such as systemic inflammation and gut-derived metabolites influence BDNF expression through epigenetic mechanisms. Butyrate, a microbial short-chain fatty acid and endogenous HDAC inhibitor, enhances histone acetylation at BDNF loci, restores synaptic plasticity, and exerts antidepressant-like effects in stress-exposed maternal models. These findings provide a mechanistic link between microbiota-driven epigenetic regulation, neuroplasticity, and PPD vulnerability (43–45).

*Epigenetic modulation of oxytocin signaling and maternal bonding*: Oxytocinergic signaling is central to maternal behavior, social reward processing, and mother-infant attachment processes that are frequently disrupted in PPD (6). Epigenetic alterations of the OXTR gene, particularly increased promoter methylation, have been associated with reduced oxytocin receptor expression in limbic regions such as the amygdala and nucleus accumbens (6). Clinical studies demonstrate that higher OXTR methylation in postpartum women correlates with impaired maternal sensitivity, reduced bonding behaviors, and increased depressive symptom severity (6, 46). Furthermore, non-coding RNAs (ncRNAs), including stress-responsive microRNAs and RNA epigenetic regulators, fine-tune oxytocin signaling and synaptic plasticity in maternal neural circuits. Dysregulated expression of miRNAs targeting OXTR, synaptic scaffolding proteins (5, 6), and plasticity-related transcripts has been reported in postpartum mood disorders, suggesting an additional epigenetic layer influencing maternal bonding and emotional regulation (46).

*PPD-specific epigenetic biomarkers*: Several epigenetic biomarkers with potential clinical relevance for PPD have been identified. These include differential methylation of immune and stress-response genes such as IL6, TNFA, CRP, NR3C1, FKBP5, and OXTR, collectively reflecting altered neuroimmune–endocrine crosstalk during the postpartum period. Epigenome-wide association studies (EWAS) have identified reproducible antenatal DNA methylation signatures most notably in HP1BP3 and TTC9B that predict postpartum depressive symptoms months after delivery (40–42). More recent integrative epigenomic analyses further demonstrate that PPD exhibits a partially distinct molecular signature compared with major depressive disorder, emphasizing the importance of reproductive hormonal context in shaping disease-specific epigenetic mechanisms (40, 41, 47).

### Innervated autonomic nervous system and the ‘small brain’: microbiome in postpartum depression (PPD)

Gut microbiome is a highly diverse ecosystem, historically believed to be predominantly bacterial in nature. However, emerging research has demonstrated that the microbiome also includes viruses, fungi, protozoa, and helminths, all of which contribute to host physiology and health (48). Among the dominant bacterial phyla colonizing the human gut, Bacteroidetes, Firmicutes, Actinobacteria, Fusobacteria, and Proteobacteria play critical roles in metabolism and immune function (48, 49). These microorganisms process complex dietary components, generating essential metabolites such as SCFAs, vitamins, amino acids, trimethylamine-N-oxide (TMAO), and lipopolysaccharides (LPS), which influence systemic physiology.

### Gut microbial influence on neurotransmitter production

A crucial aspect of gut microbiome functionality is its impact on neurotransmitter synthesis. Specific bacterial taxa contribute to the production of key neurotransmitters that regulate neurocognitive processes. Notably: *Serotonin*: Candida, Escherichia, Enterococcus, and Streptococcus influence serotonin production via tryptophan metabolism (50). *Gamma-Aminobutyric Acid (GABA)*: Bifidobacterium and Lactobacillus are involved in GABA synthesis, which plays a role in inhibitory neurotransmission and mood regulation (50). *Dopamine*: Bacillus and Serratia contribute to dopamine production through their impact on amino acid metabolism (50). *Norepinephrine*: Certain fungal species, such as *Saccharomyces boulardii*, have been implicated in norepinephrine biosynthesis (50). These microbial-derived neurotransmitters do not function in isolation but participate in a dynamic crosstalk between the gut and brain through endocrine, immune, and neural pathways. Such interactions are integral to the gut-brain axis, influencing mood, cognition, and susceptibility to neuropsychiatric disorders, including postpartum depression (PPD) (51–53). Similarly, within the gut microbiota, interspecies communication through autoinducer-2 (AI-2) has been shown to modulate microbial composition, favoring Firmicutes over Bacteroidetes following antibiotic-induced dysbiosis (54). Given the critical role of microbial balance in mental health, microbial communication may contribute to neuropsychiatric disorders by altering microbial-derived metabolite production and immune signaling (54).

### Blood–brain barrier: a regulatory interface in gut-brain communication

The blood–brain barrier (BBB) serves as a selective barrier that restricts the direct entry of circulating molecules, including gut-derived neurotransmitters, into CNS (54, 55). Structurally, the BBB consists of three primary layers: Endothelial cell layer forms the innermost lining of brain capillaries, with tight junctions that prevent paracellular transport (55, 56) whereas basement membrane provides structural support and contains extracellular matrix components along with peripheral cells (51–53). Astrocyte endfeet and pericytes are comprised of the outer layer, modulating BBB permeability and neurovascular coupling (51–53). Despite its restrictive nature, the BBB permits selective transport of specific neuroactive compounds. For instance, gamma-aminobutyric acid (GABA) can cross the BBB via specialized transporters, allowing gut-derived GABA to exert neurological effects (50, 51). However, for most neurotransmitters, their influence on the brain is indirect, occurring through gut-brain signaling pathways that involve the enteric nervous system, immune modulation, and vagal nerve activation (50, 51).

Disruptions in gut microbiota composition, microbial metabolite production, and BBB integrity have been increasingly linked to postpartum depression. The gut microbiome’s influence on neurotransmitter synthesis, immune responses, and neural plasticity underscores its role in mood disorders. Research suggests that microbial dysbiosis during pregnancy and postpartum may contribute to altered neuroinflammatory pathways, hormone regulation, and epigenetic modifications associated with depressive symptoms. Further investigation into the gut-brain axis and microbiome-targeted interventions holds promise for novel therapeutic strategies in managing PPD (2).

### Genetics and epigenetics of postpartum depression (PPD) and gut microbiota

As we discussed above, microbiota across gut plays a fundamental role in modulating gastrointestinal, hormonal, immune, and neural homeostasis, influencing various physiological and psychological processes (57). Microbiota-gut-brain axis introduced to explore the bidirectional interaction between gut microbiota and the brain, particularly in psychiatric disorders such as PPD (58). Oral administration of *Lactobacillus* has been shown to suppress pro-inflammatory cytokines and typically enhance brain-derived neurotrophic factor (BDNF) expression across hippocampus, leading to anxiolytic and antidepressant-like effects in murine models (57).

### Dysbiosis in depression: a microbial perspective

Comparative studies between individuals with depression and healthy controls reveal significant differences in gut microbiota composition, including alterations in the specific bacterial flora presence and shifts in the changes in overall microbial community structure (59–61). Certain microbial flora correlates with anxiety and depressive symptoms, while others appear to confer resilience against depression. For instance, major depressive disorder is associated with a higher presence of bacterial taxa such as the Firmicutes (58, 62). These microbial alterations influence depression via mechanisms including inflammation modulation, neurotransmitter synthesis, and gut-brain signaling, elucidating the microbiota’s role in neuropsychiatric health. Despite differences between human and murine microbiomes, evidence consistently supports the microbiota’s role in anxiety and depression-related behaviors (63).

Mendelian randomization is a statistical approach that leverages genetic variation to ascertain causal relationships between microbiome across the gut as well as PPD, reducing potential confounding factors (64, 65). According to previous reports, Mibiogen database serves as a bioinformatics server enabling researchers to investigate the gut microbiota’s causal role in various ailments such as eclampsia (66, 67), pregnancy-related difficulties (67), as well as ischemic stroke (68). MR analysis applied to gut microbiota GWAS, which could enable to ascertain causal relationship between specific bacterial features and PPD. This finding strengthens the conceptual framework of the MGB axis and highlights the shared genetic underpinnings of GI and psychiatric ailments, as confirmed through comprehensive genomic analyses (69).

## Key bacterial genera associated with PPD

### Actinobacteria: a potential protective role

Actinobacteria, a dominant phylum in the gut microbiota, has been implicated in depression resilience. Tian et al. demonstrated that the presence of Actinobacteria (such as *Bifidobacterium* and *Corynebacterium*) was significantly higher in healthy control mice than those with PPD (70). Similarly, Jiang’s high-throughput sequencing related to 46 depressed patients and 30 healthy controls demonstrated a greater presence of Actinobacteria and Firmicutes among healthy cohort (71). Furthermore, MR studies indicate that Actinobacteria may exert a protective effect against MDD (72). Actinobacteria’s potential neuroprotective effects may be mediated through its ability to compete with pathogenic bacteria for nutrients as well as adhesion regions, thus, maintaining microbial balance. Moreover, Actinobacteria could produce crucial antibiotics such as penicillin, tetracycline, and erythromycin (73). This antibiotic production may inhibit pathogen colonization and support immune homeostasis. Additionally, some Actinobacteria species regulate host immune responses, further stabilizing gut-brain interactions. While these findings are promising, further studies is required to delineate the precise mechanisms through which Actinobacteria mitigate PPD symptoms.

### *Holdemanella*: a novel target in depression research

*Holdemanella* genus has emerged as a potential modulator of depression risk. In a study examining post-stroke depression (PSD), fecal samples from 232 acute ischemic stroke patients were analyzed using 16S rRNA sequencing. Hamilton Depression Rating Scale (HAMD-3) assessment revealed a significant reduction in overall presence of *Holdemanella* in PSD patients, with a negative correlation between *Holdemanella* levels and depression severity (74). These findings suggest that increasing *Holdemanella* abundance may have a protective effect against depressive symptoms. Additional studies corroborate these findings, demonstrating that higher *Holdemanella* levels correlate with lower depression incidence (75, 76). Interestingly, omega-3 fatty acid deficiencies are commonly observed in depressed individuals (77). While the efficacy of omega-3 supplementation in alleviating depression remains inconclusive, dietary intake of omega-3-rich fish has been associated with a higher *Holdemanella* abundance (71). This raises the intriguing possibility that modulating gut microbiota composition through dietary interventions, such as omega-3 supplementation, may serve as a novel strategy for PPD prevention and treatment.

Despite its valuable insights, this study presents certain limitations. GWAS meta-analysis related to microbiome across the gut included both men and women participants, raising potential concerns regarding gender-related biases. Although genetic variants on sex chromosomes were subjected to exclusion and adjustments pertinent to gender were implemented, residual confounding cannot be ruled out (78). Future research should prioritize subgroup analyses to explore the influence of gender and genetic diversity on gut microbiota-PPD associations. Further investigations utilizing metagenomic analysis and functional experiments are warranted to elucidate the mechanistic pathways through which gut microbiota influences PPD. A deeper understanding of these interactions will pave the way for precision-targeted interventions, enhancing therapeutic strategies for PPD and related mood disorders. By integrating genomic, microbial, and neurobiological perspectives, future studies can provide a clear understanding pertinent to intricate relationship between gut microbiota and mental health, ultimately contributing to improved maternal well-being and psychiatric care (79).

### Epigenetics of PPD and implications of gut-microbiome

Epigenetics encompasses dynamic and heritable alterations in gene expression which occur independently of changes in the underlying DNA elements (80, 81). These modifications such as DNA methylation and hydroxymethylation, histone proteins alterations by post-translational changes, chromatin remodeling, and diverse RNA modifications. These intricate and interdependent mechanisms serve as molecular mediators that shape cellular responses to environmental and physiological stimuli (81, 82). Epigenetic mechanisms contribute to both direct modifications in gene expression and gene–environment interactions, thereby playing a crucial role in the pathogenesis of complex disorders including depression and PPD (83–85). Growing evidence describes the significant role of epigenetic alterations in modulating multiple biological pathways implicated in the etiology of major depressive disorder (MDD) and other stress-related psychiatric conditions, including anxiety disorders and PTSD (86–88). Epigenetic modifications influence neuronal plasticity, synaptic remodeling, and memory consolidation by regulating gene transcription and chromatin accessibility (89–92). In preclinical models of MDD and related disorders, aberrant epigenetic regulation is linked to dysregulated stress response systems, disrupted monoaminergic neurotransmission, and heightened neuroinflammatory signaling, all of which contribute to disease pathophysiology (93, 94).

Recent studies have highlighted the role of heterogeneous nuclear ribonucleoprotein A2B1 (HNRNPA2B1) as a potential biomarker for postpartum depression, suggesting its diagnostic relevance in psychiatric disorders (7). Additionally, the RNA-binding protein YTH N6-methyladenosine RNA-binding protein 1 (YTHDF1) plays a pivotal role in synaptic plasticity and cognitive functions by enhancing the translation of specific neuronal transcripts across hippocampus. Loss of YTHDF1 expression impairs synaptic transmission and long-term potentiation, resulting in damage to learning and memory, which are hallmark features of depression-related cognitive dysfunction (95).

While N6-methyladenosine (m6A) RNA modifications have been extensively studied in depression, other RNA modifications include N4-acetylcytidine (ac4C) and 5-methylcytosine (m5C), remain largely unexplored in the context of mood disorders. The enzyme N-acetyltransferase 10 (NAT10), a member of GNAT family, facilitates ac4C modification on thymidine residues, thereby modulating mRNA translation efficiency (96). In a mouse model subjected to chronic mild stress, NAT10 expression was found to be significantly upregulated in the hippocampus, correlating with anxiety and depression-like behaviors. Pharmacological inhibition of NAT10 activity resulted in the attenuation of these depressive phenotypes, suggesting its potential as a therapeutic target (97).

Among various RNA modifications, m5C is one of the most abundant, occurring on tRNA and rRNA, where it is enzymatically catalyzed by members of the NOL1/NOP2/SUN domain (NSUN) family and DNA methyltransferase homolog DNMT2. Dysregulation of cortical NSUN2, a tRNA methyltransferase, disrupts tRNA methylation patterns and alters the translational efficiency of glycine-rich synaptic proteins, leading to profound effects on synaptic homeostasis and depressive behaviors in murine models. These findings underscore the importance of RNA epigenetics in the molecular underpinnings of depression and open new avenues for developing RNA-targeted interventions in psychiatric disorders (98).

### Fetal programming and epigenetic foundations: PPD

Ensuring adequate nutrient supply to the developing fetus is essential for normal physiological and neurodevelopmental processes. Disruptions in nutrient availability can induce compensatory mechanisms in embryonic and fetal cells that, while adaptive in the short term, may lead to persistent metabolic dysregulation postpartum (99). This concept underlies the “fetal origins hypothesis,” initially proposed by David J. Barker, which suggests that environmental stressors during fetal development particularly nutritional deficiencies can predispose individuals to chronic diseases such as metabolic syndrome later in life (100–102). Subsequent refinements of this hypothesis have expanded its scope to include maternal behaviors (e.g., smoking, obesity, and alcohol intake) and socioeconomic influences (e.g., pollution, educational disparities, and chronic stress), all of which shape fetal epigenetic programming and long-term health trajectories (103–108).

Fetal metabolic imprinting, a central concept within this framework, suggests that metabolic adaptations established in utero persist into adulthood through epigenetic modifications. The field of epigenetics has elucidated various molecular mechanisms underlying these phenomena, providing evidence that environmentally induced changes in gene expression are mediated through alterations in chromatin architecture rather than DNA sequence modifications (109). These modifications, which are heritable and potentially transgenerational, serve as key regulators of gene expression patterns in response to environmental exposures (110).

Among the most extensively studied epigenetic mechanisms is DNA methylation, which involves the covalent attachment of a methyl group to the fifth carbon of cytosine (5mC) within CpG dinucleotides. This modification, catalyzed by DNA methyltransferase (DNMT) enzymes, plays a crucial role in modulating gene transcription (111–117). Typically, hypermethylation in gene promoter regions leads to transcriptional repression, whereas increased methylation within gene bodies is often associated with enhanced gene expression (118, 119). The process of DNA methylation undergoes extensive reprogramming during embryonic development, where both maternal and paternal methylation marks are erased and re-established through a tightly regulated process influenced by genetic, dietary, and environmental factors (120–123).

DNA methylation patterns can be shaped by both fixed genetic determinants, such as single nucleotide polymorphisms (SNPs) affecting one-carbon metabolism enzymes, and modifiable factors, including dietary intake (124). Nutritional components such as choline, folic acid, betaine, and vitamin B12 are essential methyl donors required for maintaining DNA methylation homeostasis. Prolonged deficiencies in these nutrients can result in aberrant 5mC patterns, potentially altering gene expression profiles linked to neurodevelopment and metabolic function (125, 126). Despite advancements in public health initiatives, suboptimal micronutrient intake remains prevalent worldwide, affecting a significant portion of the population, including pregnant women and children (127, 128). Nearly two billion individuals suffer from micronutrient deficiencies, with potential consequences ranging from impaired immune responses to neurocognitive deficits (4, 129).

A recent investigation (7) sought to unravel the molecular basis of PPD by identifying key genetic contributors and elucidating epigenetic mechanisms underlying its pathogenesis. The study employed weighted gene co-expression network analysis (WGCNA) to examine differentially expressed genes (DEGs), aiming to identify gene modules exhibiting significant associations with PPD. Among the identified modules, the three most relevant were selected based on their correlation with the disorder. Hub genes within these modules were further refined through rigorous cross-validation techniques to enhance the robustness of candidate gene selection (7). In parallel, methylation profiling from the GSE44132 dataset was analyzed to uncover epigenetic modifications that may contribute to PPD susceptibility.

The analysis identified 8,979 DEGs, which were subsequently subjected to WGCNA. Among the generated co-expression modules, a few genes demonstrated the highest correlation with PPD onset. Functional enrichment analysis based on KEGG pathways revealed that hub genes in these modules were predominantly associated with the neurotrophin signaling cascade, chemokine-mediated immune responses, Fcγ receptor-mediated phagocytosis, and MAPK signaling network, all of which have been implicated in neuroinflammation and stress response regulation (7). Through cross-validation, eight hub genes such as HNRNPA2B1, IL10, RAD51, UBA52, NHP2, RPL13A, FBL, and SPI1 were identified as having significant potential in distinguishing individuals with PPD. Functional insights gained from GSEA further highlighted the involvement of pathways related to olfactory signal transduction, butanoate metabolism, melanoma-associated processes, aminoacyl-tRNA biosynthesis, and lysine catabolism, suggesting a multifaceted interplay between metabolic, immune, and neurobiological pathways in PPD pathophysiology (7).

Furthermore, methylation analysis revealed that aberrant DNA methylation patterns in key genes such as HNRNPA2B1, RPL13A, and UBA52 were significantly correlated with PPD vulnerability. These findings provide a deeper understanding of the transcriptomic and epigenetic landscape of PPD, reinforcing the notion that complex gene–environment interactions contribute to its etiology. Moreover, the identification of epigenetically modified genes opens new avenues for targeted therapeutic interventions, offering the prospect of precision medicine approaches for PPD management (7).

### Gut microbiota and nutrient bioavailability: post-partum depression

While dietary intake influences nutrient availability, circulating micronutrient levels are also modulated by gut microbiota, which play a pivotal role in nutrient extraction, vitamin synthesis, and metabolic regulation (130–132). Composition and functional capacity of the gut microbiome directly impact the bioavailability of both macro- and micronutrients (130, 131, 133–140). The beneficial health effects of whole grains, vegetables, and fruits are largely attributed to microbial fermentation byproducts, such as SCFAs like butyrate, and bioactive phenolic compounds like protocatechuic acid (141–145). However, gut microbial metabolism can also exert detrimental effects on host health by transforming essential nutrients into harmful metabolites. For instance, bacterial choline metabolism occurs through choline utilization that in turn generates trimethylamine (TMA), reducing choline bioavailability (130). TMA is subsequently oxidized by hepatic flavin-containing monooxygenase (FMO) enzymes into trimethylamine-N-oxide (TMAO), a metabolite implicated in metabolic disorders (146–150).

Choline availability, which is essential for placental macronutrient transport and fetal growth, is also critical for maternal mental health. Choline is a precursor for acetylcholine, a neurotransmitter involved in mood regulation, and phosphatidylcholine, a key component of neuronal membranes (99, 151, 152). Insufficient choline intake during pregnancy and postpartum may lead to neurochemical imbalances, contributing to the development of PPD. Studies suggest that choline deficiency is linked to increased HPA axis dysregulation, leading to elevated cortisol levels, which are commonly observed in women with PPD (99, 151, 152). Furthermore, low choline levels may impair the synthesis of S-adenosylmethionine (SAM), a methyl donor required for neurotransmitter biosynthesis and epigenetic regulation of mood-related genes (99, 151, 152).

Animal studies have demonstrated that maternal high-fat (HF) diets exacerbate microbial choline depletion, leading to a 20% reduction in fetal choline levels and concurrent increases in fetal adiposity (151, 153, 154). These effects stem from heightened hepatic demand for phosphatidylcholine (PtdCho), which is required for VLDL synthesis and lipid transport (155). Impairments in PtdCho synthesis contribute to hepatic steatosis, a pathological condition characterized by triglyceride accumulation in liver tissue (156). Notably, disruptions in maternal choline metabolism, exacerbated by gut microbial activity, may predispose offspring to metabolic dysfunction and altered lipid homeostasis (131, 157). However, these metabolic disturbances may also extend to maternal health, as postpartum metabolic dysregulation has been associated with an increased risk of mood disorders, including PPD.

Although choline supplementation in murine models has been shown to normalize placental and fetal growth parameters, as well as mitigate excessive lipid accumulation in fetal hepatic tissue (151, 154), the optimal choline intake required to achieve similar benefits in humans remains uncertain. Pregnancy and lactation significantly increase maternal choline demands, with fetal choline concentrations in amniotic fluid being 14-fold higher than maternal plasma levels (158, 159). Alarmingly, epidemiological data indicate that 90–95% of pregnant women fail to meet recommended choline intake levels, even with prenatal care (160). Additionally, nearly half of all women lack the ability to induce endogenous choline biosynthesis during pregnancy, further necessitating dietary supplementation (161). Given the emerging evidence linking choline insufficiency to increased stress responsivity and PPD risk, addressing maternal choline intake through diet or supplementation may offer a novel strategy to mitigate postpartum mood disturbances (4).

### Integrated neuroimmune mechanisms linking microglia, cytokines, gut dysbiosis, and PPD

Although inflammation was previously mentioned only superficially, it is increasingly clear that neuroimmune interactions constitute a core mechanistic pathway in PPD, linking systemic immune activation, gut-brain signals, and central nervous system responses.

*Microglial priming during pregnancy and the postpartum period*: Microglia are the resident immune cells of the central nervous system and are highly sensitive to both systemic immune signals and peripheral metabolic cues. During pregnancy, extensive hormonal, immunological, and microbiota shifts set the stage for altered microglial reactivity postpartum. Priming refers to a state wherein microglia become hypersensitive to subsequent inflammatory signals, lowering their activation threshold and amplifying neuroinflammatory responses (162–164). In models of stress and dysbiosis, microglial priming impairs hippocampal neurogenesis and fosters stress vulnerability with key features implicated in mood disorders (162–165). This primed state can persist through the peripartum period, making the maternal brain more susceptible to secondary inflammatory stimuli that disrupt synaptic plasticity and mood regulation (162–166). Pregnancy itself involves dynamic microglial remodeling, and abrupt postpartum shifts in immune status including changes in cytokines and endocrine signals that can trigger microglial activation pathways that alter neural circuit function underlying emotion and reward (162–166). Elevated numbers of activated microglia and increased expression of pro-inflammatory markers such as NF-κB, TNF-*α*, and IL-6 have been observed in preclinical models exposed to prenatal depression-associated microbiota, supporting a direct link between immune priming and depressive phenotypes (162–166).

*Cytokine-mediated epigenetic remodeling in maternal brain circuits*: Pro-inflammatory cytokines such as IL-6, TNF-α, and IL-1β not only modulate microglial activation but also influence gene expression through epigenetic mechanisms. Cytokines can recruit chromatin modifiers, alter DNA methylation patterns, and change histone acetylation states in stress-responsive neural circuits (45, 162–171). For example, NF-κB signaling potently activated by peripheral inflammatory signals interacts with histone acetyltransferases and deacetylases, driving epigenetic changes that affect expression of neurotrophins, glucocorticoid receptors, and mood-related genes. Elevated circulating pro-inflammatory cytokines observed in maternal inflammation can thereby exert lasting effects on chromatin accessibility in hippocampal and prefrontal regions implicated in mood regulation and maternal behavioral responsiveness (162–167). This cytokine-driven epigenetic remodeling provides a biologically plausible mechanism linking immune activation to persistent changes in neural circuit function, offering a pathway by which perinatal inflammation can engrain depressed mood states beyond the immediate immune response.

*How gut dysbiosis drives neuroinflammation in PPD*: Multiple high-quality studies now demonstrate that gut microbiota dysbiosis is a critical upstream driver of systemic and central inflammation that contributes to depressive phenotypes, including those relevant to PPD. Dysbiosis typically defined by reduced diversity and depletion of SCFA-producing taxa leads to increased gut permeability (“leaky gut”), facilitating translocation of bacterial endotoxins such as lipopolysaccharide (LPS) into the circulation. Elevated LPS and microbial-associated molecular patterns trigger systemic immune activation, including TLR-4/NF-κB and inflammasome pathways, which subsequently compromise blood–brain barrier integrity and allow peripheral cytokines to influence the CNS directly (162–167). In PPD-relevant models, microbiota dysbiosis induces depression-like behavior through hippocampal NLRP3 inflammasome-mediated neuroinflammation, which can be reversed by fecal microbiota transplantation from healthy donors. These findings provide a causal link between gut microbial composition, innate immune activation, and central neuroinflammation in postpartum mood disruption (162–167).

Mechanistically, microbial metabolites such as SCFAs and tryptophan catabolites can also modulate both peripheral and central immune function. SCFAs generally exert anti-inflammatory effects, whereas an imbalance favoring pro-inflammatory metabolites increases expression of IL-1β, IL-6, TNF-*α*, and other cytokines that activate microglia (162–167). Altered SCFA profiles have been linked to depressive symptoms and inflammatory markers in clinical populations, demonstrating the importance of microbiota-immune interactions in mood regulation. Therefore, a significant integration of microglial priming, cytokine-driven epigenetic remodeling, and gut dysbiosis-triggered systemic inflammation constructs a biologically plausible, evidence-based neuroimmune framework for PPD. This framework not only explains how immune signals alter central pathways governing mood and maternal behavior but also highlights potential targets for intervention, including microbiota modulation, anti-inflammatory strategies, and epigenetically informed therapeutics (162–167).

### Choline deficiency, epigenetic reprogramming, and postpartum depression

Choline plays a crucial role in DNA methylation by serving as a precursor SAM, the universal methyl donor required for DNMT activity (172). Deficiencies in choline and related methyl donors result in aberrant epigenetic programming, with potential consequences for neurodevelopmental and neuropsychiatric outcomes (111–113, 116). Importantly, these epigenetic alterations may extend beyond fetal development and contribute to maternal mental health, including PPD. Choline deprivation, whether due to dietary inadequacy or excessive microbial utilization, disrupts mitochondrial function and enhances ROS production (131, 134). Increased oxidative stress has been strongly linked to neuroinflammation, a critical factor in the pathophysiology of depression (173, 174). Elevated TMAO levels further exacerbate oxidative stress, promoting DNA damage and aberrant base excision repair, which can lead to global DNA hypomethylation (173, 174).

Epigenetic dysregulation is increasingly recognized as a key factor in neurodevelopmental disorders, including depression, anxiety, schizophrenia, and Alzheimer’s disease (175). Studies in murine models have demonstrated that maternal choline deficiency leads to reduced global DNA methylation in fetal brain tissues (4, 176), suggesting a potential mechanism linking early-life nutritional status to cognitive and behavioral outcomes. These epigenetic modifications may also influence maternal postpartum mental health by altering the expression of genes involved in stress regulation, neurogenesis, and inflammatory pathways. Notably, similar reductions in DNA methylation have been observed in neonates born to mothers harboring high levels of choline-depleting gut bacteria, despite adequate dietary choline intake (4, 131). Given that maternal gut dysbiosis is associated with increased systemic inflammation and altered neurotransmitter metabolism, it is plausible that disruptions in microbial choline metabolism could contribute to the onset of PPD. Furthermore, offspring of mothers with dysbiotic gut microbiota exhibit altered behavioral phenotypes, underscoring the complex interplay between maternal diet, gut microbiome, and neurodevelopmental programming (4, 131).

Current choline intake recommendations do not consider interindividual genetic variability, such as single nucleotide polymorphisms in genes involved in endogenous choline biosynthesis (4, 177, 178). Additionally, microbial metabolism of choline can lead to biochemical signatures indicative of choline deficiency (4, 176), potentially placing the majority of pregnant women at risk of inadequate choline availability. This deficiency may have profound implications not only on fetal development and epigenetic modifications but also on maternal mental health, as choline-dependent pathways regulate stress response and mood-related neurotransmitter synthesis. The urgent need for personalized nutritional strategies during pregnancy is underscored by accumulating evidence from murine and human studies, suggesting that diminished choline bioavailability influences fetal programming through epigenetic pathways. Beyond choline metabolism, the gut microbiome is now recognized for its role in transgenerational epigenetic inheritance. A study on infants at 6 months of age revealed that maternal gut microbiota composition particularly Firmicutes-dominant versus Bacteroidetes-dominant profiles correlated with differential DNA methylation patterns (4, 179). Notably, genes differentially methylated in infants born to mothers with elevated Firmicutes levels were associated with metabolic disorders, inflammation, and lipid metabolism, reinforcing the role of microbial-derived metabolites in shaping long-term health trajectories (4). These findings raise the possibility that gut microbiota-mediated epigenetic programming may also impact maternal susceptibility to PPD by modulating inflammatory and neuroendocrine pathways (4).

### Microbial metabolism, epigenetic regulation, and postpartum depression

Negative impact of microbial activity extends beyond nutrient depletion to the accumulation of metabolic byproducts, such as TMAO. TMAO has been implicated in various metabolic and cardiovascular diseases (4, 148–150). Recent studies suggest that elevated TMAO levels may also play a role in neuroinflammation and oxidative stress, both of which are key contributors to mood disorders, including PPD. Maternal gut dysbiosis during pregnancy may lead to increased microbial choline metabolism (4, 148–150), reducing choline bioavailability and contributing to the epigenetic modifications implicated in depression. However, more research is necessary to delineate the precise consequences of microbial metabolism during pregnancy and early infancy (Table 1).

**Table 1:** Integrating microbiome, genetics, epigenetics, and their impact on postpartum depression (PPD).

Microbiome/mediators	Mechanisms of genetics/epigenetics to modulate PPD	Signaling genes involved	Refs
Lactobacillus	Suppresses pro-inflammatory cytokines; enhances BDNF expression; modulates gut-brain axis	BDNF (Brain-Derived Neurotrophic Factor)	(57)
Firmicutes	Altered abundance linked to depressive symptoms via inflammation and neurotransmitter synthesis	Not specified	(58, 62)
Actinobacteria (e.g., Bifidobacterium, Corynebacterium)	Competes with pathogenic bacteria; produces antibiotics; regulates immune response, stabilizing gut-brain interactions	Not specified	(70, 71–73)
Holdemanella	Lower abundance correlates with increased depression severity; may be influenced by omega-3 fatty acids	Not specified	(71, 74–77)
Gut Microbiota (General)	Mendelian randomization (MR) analysis reveals shared genetic underpinnings between microbiota and psychiatric disorders	Various bacterial GWAS loci	(49–54)
HNRNPA2B1	Identified as a potential biomarker for PPD	HNRNPA2B1	(7)
YTHDF1	Regulates synaptic plasticity and cognitive functions by enhancing neuronal transcript translation	YTHDF1	(95)
NAT10	Modulates RNA acetylation (ac4C modification); inhibition mitigates depression-like behaviors	NAT10	(96, 97)
NSUN2	Affects tRNA methylation, influencing synaptic homeostasis and depressive behaviors	NSUN2, DNMT2	(98)
Epigenetic DNA Methylation (General)	Regulates gene transcription via DNMTs; influences fetal programming of metabolic and neurodevelopmental pathways	DNMTs, CpG methylation	(111–123)
Dietary Methyl Donors (Choline, folic acid, betaine, vitamin B12)	Maintain DNA methylation homeostasis; deficiencies cause aberrant 5mC patterns affecting neurodevelopment	5mC methylation	(124–126)
Gut Microbiota & Nutrient bioavailability	Microbiota modulates absorption and synthesis of key vitamins and methyl donors, influencing epigenetic programming	Not specified	(130–140)
Choline & One-carbon Metabolism	Essential for SAM synthesis, neurotransmitter production; deficiency linked to HPA axis dysregulation & PPD risk	SAM, BDNF, DNMTs	(99, 151, 152)
TMAO-producing bacteria	Converts choline to TMAO, reducing bioavailability; linked to oxidative stress, neuroinflammation, and mood disorders	FMO enzymes	(146, 148–150)
Maternal High-fat diet & microbiota	Alters microbial metabolism, reducing fetal choline and increasing oxidative stress; linked to metabolic dysfunction and PPD	Not specified	(151, 153, 154)
SCFAs (short-chain fatty acids)	Modulate histone acetylation and methylation, influencing stress resilience and neurotransmitter regulation	BDNF, HDACs	(4, 197)
Microbiota and transgenerational epigenetics	Microbial composition influences DNA methylation patterns in offspring, affecting inflammation and metabolic pathways	Various methylation loci	(4, 179)
Antibiotic-induced gut dysbiosis	Disrupts microbiota homeostasis, increasing inflammation and HPA axis dysregulation; potential epigenetic effects	Cytokine genes, HPA axis regulators	(4, 190–192)
Maternal obesity and gut dysbiosis	Alters infant microbiome, SCFA profiles, and metabolic health; linked to increased PPD risk	SCFA pathways, leptin & insulin genes	(4, 193–196)
Epigenetic effects of SCFAs	SCFAs influence histone modifications and stress resilience; butyrate modulates BDNF expression	BDNF, HDACs	(4, 197)

### Mechanistic links between gut microbiota-derived metabolites and epigenetic regulation in PPD

**Figure 2:**
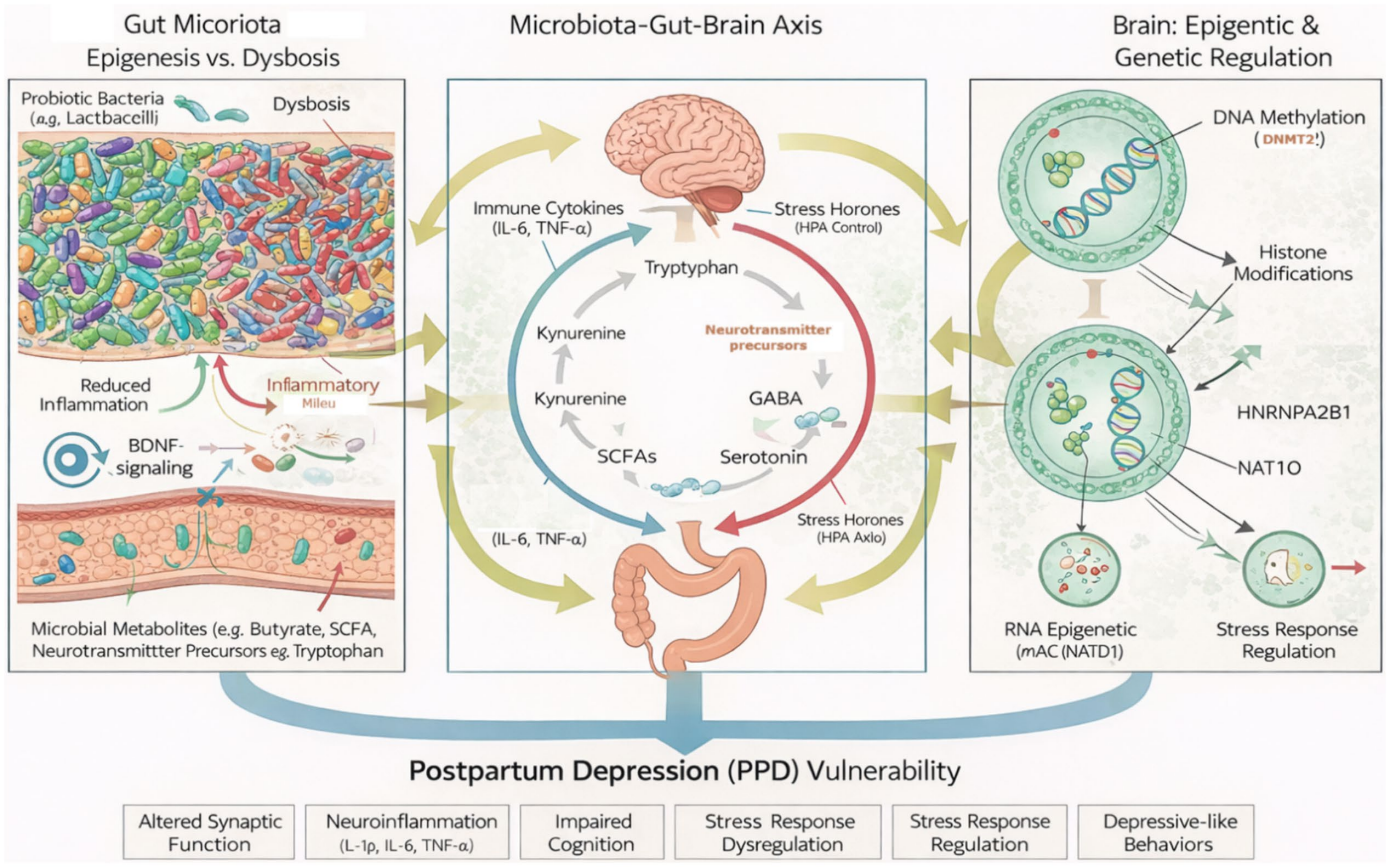
Interconnecting gut microbiota-immune-epigenetic pathways contributing to postpartum depression (PPD). The figure illustrates the multi-level interactions between gut microbiota homeostasis, the microbiota-gut-brain axis, and epigenetic and genetic regulation in the brain that collectively influence vulnerability to postpartum depression. *Left panel (Gut microbiota homeostasis vs. dysbiosis)*: A balanced gut microbiota enriched with beneficial bacteria (e.g., *Lactobacillus* and Firmicutes) supports intestinal barrier integrity, produces microbial metabolites such as short-chain fatty acids (SCFAs), and supplies neurotransmitter precursors (e.g., tryptophan). These effects are associated with reduced inflammation and enhanced brain-derived neurotrophic factor (BDNF) signaling. In contrast, gut dysbiosis is characterized by increased intestinal permeability, elevated inflammatory mediators (e.g., lipopolysaccharides), and disrupted microbial metabolite production, contributing to systemic inflammation and altered neurotrophic signaling. *Central panel (Microbiota-gut-brain axis)*: Bidirectional communication between the gut and brain occurs through immune signaling (e.g., IL-6, TNF-*α*), vagal nerve pathways, stress hormones (e.g., cortisol), and metabolic intermediates. Alterations in tryptophan metabolism toward the kynurenine pathway promote neuroinflammation and excitotoxicity, while dysregulated cytokine signaling further modulates neurotransmitter synthesis and stress-response pathways relevant to PPD. *Right panel (Brain epigenetic and genetic regulation)*: Neuronal nuclei depict chromatin-level regulation involving DNA methylation (DNMT2), histone modifications (acetylation and methylation), chromatin remodeling, and RNA epigenetic modifications (including m^6^A, ac⁴C, and m^5^C). Key molecular regulators such as HNRNPA2B1, YTHDF1, NAT10, and NSUN2 influence RNA stability, translation, synaptic plasticity, memory formation, and stress-response regulation, thereby shaping depressive-like behaviors. The convergence of gut dysbiosis, immune activation, altered neuroendocrine signaling, and epigenetic dysregulation results in impaired synaptic function, neuroinflammation, cognitive deficits, dysregulated stress responses, and increased vulnerability to postpartum depression. PPD, postpartum depression; BDNF, brain-derived neurotrophic factor; SCFAs, short-chain fatty acids; IL-6, interleukin-6; TNF-*α*, tumor necrosis factor alpha; LPS, lipopolysaccharide; HPA axis, hypothalamic–pituitary–adrenal axis; DNMT2, DNA methyltransferase 2; m^6^A, N^6^-methyladenosine; ac⁴C, N⁴-acetylcytidine; m^5^C, 5-methylcytidine; HNRNPA2B1, heterogeneous nuclear ribonucleoprotein A2/B1; YTHDF1, YTH domain family protein 1; NAT10, N-acetyltransferase 10; NSUN2, NOP2/Sun RNA methyltransferase 2; fMRI, functional magnetic resonance imaging; SCF, synaptic connectivity function.

Accumulating evidence demonstrates that the gut microbiota exerts direct control over host epigenetic programming through metabolite-mediated signaling pathways (Figures 1, 2). These microbiota-derived metabolites function as molecular intermediates linking environmental exposures (diet, inflammation, stress) to chromatin remodeling, DNA methylation, and transcriptional regulation in neural and immune circuits implicated in PPD. Three major mechanistic axes such as short-chain fatty acids (SCFAs) (180), tryptophan-derived metabolites (181), and one-carbon metabolism (182) provide a biologically coherent framework connecting gut dysbiosis to epigenetic vulnerability during the postpartum period (45, 168–171).

### SCFAs as histone deacetylase (HDAC) inhibitors regulating neuroplasticity

Short-chain fatty acids, particularly butyrate and acetate, are produced by bacterial fermentation of dietary fiber and act as potent endogenous epigenetic regulators (183–185). Butyrate functions as a class I and II histone deacetylase (HDAC) inhibitor, increasing histone acetylation at promoter regions of genes involved in synaptic plasticity, stress resilience, and immune regulation. In the central nervous system, SCFA-mediated HDAC inhibition enhances chromatin accessibility at neurotrophic loci such as BDNF, thereby promoting hippocampal synaptic remodeling and antidepressant-like behavioral effects (183–185).

Postpartum-relevant stress and inflammatory states are associated with reduced SCFA-producing taxa, resulting in diminished HDAC inhibition and transcriptional repression of plasticity-related genes. Preclinical models demonstrate that butyrate supplementation restores histone acetylation, attenuates neuroinflammation, and normalizes stress-induced behavioral phenotypes, providing a direct mechanistic link between microbial metabolites, chromatin remodeling, and affective regulation (45, 168–171, 183–185).

### Tryptophan metabolites and aryl hydrocarbon receptor (AhR)-epigenetic signaling

Gut microbiota profoundly influence host tryptophan metabolism, diverting it toward indole derivatives (181) and the kynurenine pathway, both of which exert epigenetic and transcriptional effects through ligand-activated nuclear receptors. Microbial indoles activate the aryl hydrocarbon receptor (AhR), a transcription factor that recruits chromatin-modifying complexes to immune and neuronal gene loci. AhR signaling regulates histone acetylation and methylation patterns at genes controlling neuroinflammation, microglial activation, and stress responsivity (181, 186, 187). Dysregulated tryptophan metabolism in depressive states favors kynurenine production, increasing neurotoxic metabolites and inflammatory signaling. In postpartum contexts, altered placental and gut-derived kynurenine metabolism has been linked to increased maternal inflammation and depressive symptoms (181, 186, 187). AhR-dependent epigenetic remodeling of cytokine and neurotransmitter-related genes represents a key molecular mechanism linking microbiota-driven tryptophan metabolism to neural circuit dysfunction in PPD (181, 186, 187).

### Folate, choline, and one-carbon metabolism linking microbiota to DNA methylation

Gut microbiota contributes directly to one-carbon metabolism by synthesizing and modulating host availability of folate, choline, betaine, and other methyl donors required for S-adenosylmethionine (SAM) production and the universal methyl group donor for DNA and histone methylation (182, 188). Microbiota-dependent folate biosynthesis influences DNA methyltransferase activity and global methylation capacity, thereby shaping epigenetic landscapes in both immune cells and the brain (189). Disruption of microbial folate metabolism results in aberrant methylation of stress- and immune-response genes such as NR3C1, FKBP5, and IL6, which are implicated in PPD vulnerability. Choline and betaine metabolism further regulate methyl donor availability, linking dietary intake, microbial composition, and DNA methylation patterns in mood-related neural circuits. Human epigenome-wide association studies demonstrate that perinatal methyl donor availability predicts postpartum DNA methylation signatures associated with depressive symptom trajectories (38–42).

### Integrative relevance to postpartum depression

Collectively, these metabolite-driven epigenetic mechanisms establish a causal biological continuum linking gut microbiota composition to chromatin remodeling, transcriptional regulation, and neural circuit plasticity during the postpartum period. SCFA-mediated HDAC inhibition, AhR-dependent chromatin remodeling, and methyl donor-regulated DNA methylation converges on stress, immune, and bonding pathways that are uniquely sensitive during postpartum neuroendocrine transition. Disruption of these microbiota–epigenetic interactions provide a mechanistic basis for increased susceptibility to postpartum depression and highlights gut-targeted nutritional and microbial interventions as promising epigenetic modulators of maternal mental health (45, 168–171, 181, 183–187).

### Antibiotic exposure, gut dysbiosis, and postpartum depression risk

Perturbations in the maternal microbiome due to antibiotic exposure can have lasting intergenerational consequences. Antibiotic-induced dysbiosis in early life is associated with persistent changes in the offspring’s microbiota, affecting immune system development and neurocognitive function (4, 190). For instance, low-dose penicillin exposure during pregnancy or early life alters cytokine expression in the cortex, increases blood–brain barrier permeability, and affects behavioral outcomes in a microbiota-dependent manner (4, 191). These findings are particularly relevant to maternal mental health, as maternal gut dysbiosis has been linked to increased systemic inflammation, HPA axis dysregulation, and altered neurotransmitter production factors commonly associated with PPD. Furthermore, epigenetic mechanisms play a pivotal role in regulating inflammation, and microbial exposure in infancy (e.g., breastfeeding duration, season of birth, environmental factors) is significantly correlated with immune-related DNA methylation patterns. Interestingly, increased early-life microbial exposure is linked to reduced inflammation in adulthood (4, 192), reinforcing the importance of maintaining microbial diversity. Given that inflammation is a well-established risk factor for PPD, disruptions in maternal microbial diversity due to antibiotic exposure may predispose women to postpartum mood disturbances (4, 192).

### Influence of maternal metabolic health on infant microbiome composition and PPD

**Figure 3:**
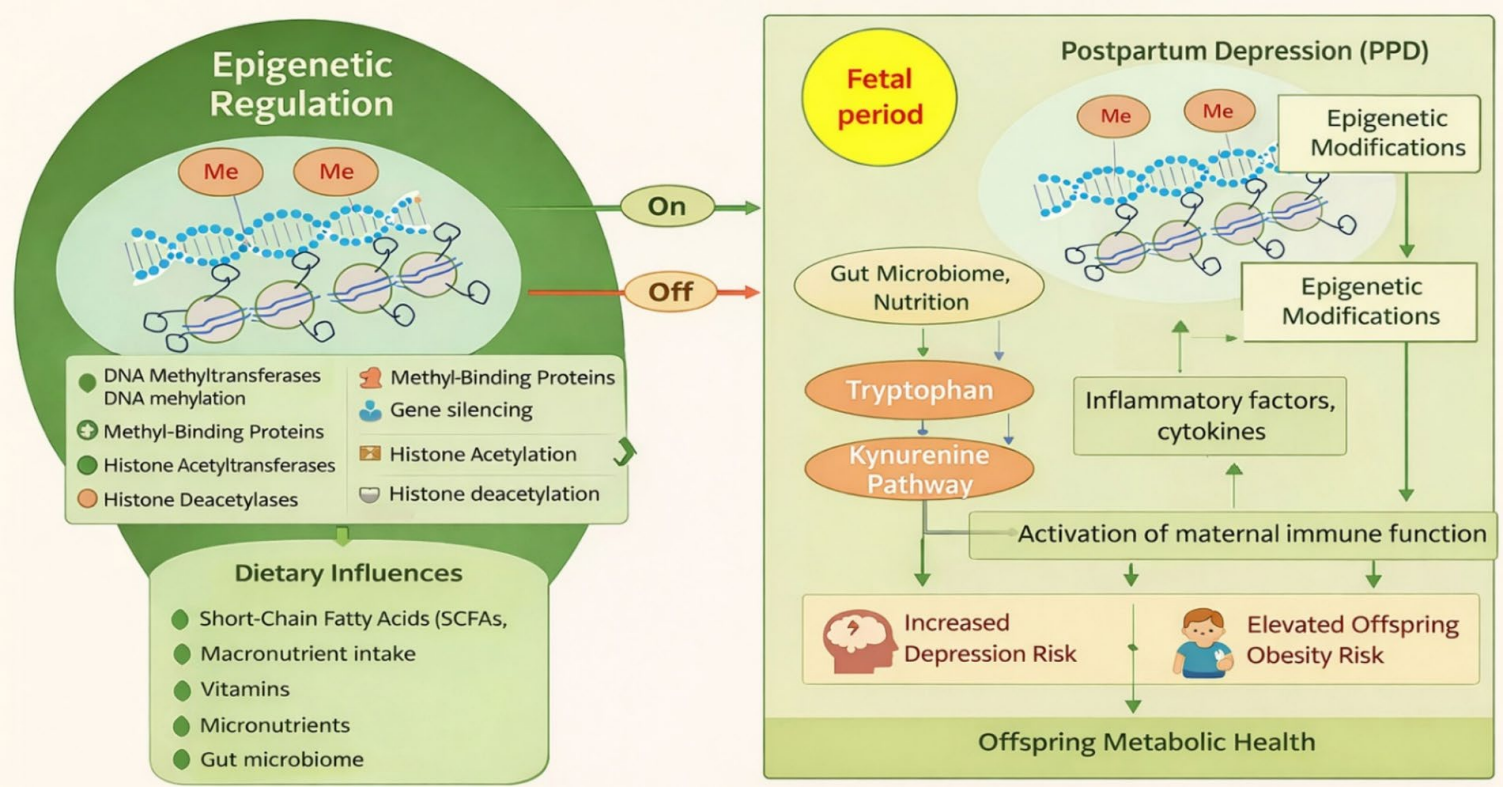
Epigenetic and immunoregulatory pathways influencing postpartum depression (PPD) and offspring metabolic health. This figure illustrates the intricate relationship between maternal diet, epigenetic modifications, and immune regulation in the context of postpartum depression. The left panel depicts key epigenetic mechanisms, including DNA methylation and histone modifications, which regulate gene expression through chromatin remodeling. Epigenetic enzymes such as DNA methyltransferases, methyl-binding proteins, histone acetyltransferases, and deacetylases contribute to these processes, affecting maternal mental health. Among various environmental influences, epigenetic factors related to diet and nutrition play a pivotal role in postpartum depression. Extensive research has highlighted the significant impact of macronutrient intake on the early-life programming of metabolic disorders. More recently, growing evidence suggests that maternal consumption of micronutrients influences offspring metabolic health later in life. Epigenetic mechanisms, including DNA methylation and histone modifications, regulate gene expression by altering chromatin structure or modulating the accessibility of genes to transcription factors. These processes are mediated by various epigenetic enzymes, such as DNA methyltransferases, methyl-binding proteins, histone acetyltransferases, and deacetylases. The interplay of these molecular modifications is increasingly recognized for its critical role in postpartum depression. Collectively, these epigenetic modifications form the epigenome, which undergoes continuous and reversible remodeling throughout an individual’s development, physiological responses, and disease progression. The kynurenine (Kyn) pathway generates metabolites with immunoregulatory properties that are essential in modulating inflammation during pregnancy. Dysregulation of this pathway has been linked to increased maternal inflammation, which, in turn, may elevate the risk of childhood obesity. Furthermore, the enzyme indoleamine 2,3-dioxygenase (IDO) is implicated in the pathophysiology of interferon-alpha-induced depression. The right panel highlights the kynurenine pathway’s role in immune regulation, emphasizing the production of metabolites that modulate inflammation during pregnancy. Dysregulated expression of kynurenine pathway enzymes in the placenta is linked to increased inflammatory responses and a higher risk of depression. The figure also elucidates the potential consequences of maternal inflammation on offspring metabolic health, suggesting that targeted interventions to reduce maternal inflammation could mitigate the risk of childhood obesity.

Beyond antibiotic use, maternal metabolic disease presents another potential source of microbiome perturbation that may persist across generations. A cohort study examining infants born to obese mothers revealed significant alterations in microbiota composition and metabolic health indicators. These infants exhibited elevated insulin and leptin levels compared to those born to normal-weight mothers, along with reduced *Gammaproteobacteria* and *Lactobacillales* abundance and altered SCFA profiles (4, 193). SCFAs, including acetate, propionate, and butyrate, play crucial roles in modulating metabolism, inflammation, and gut homeostasis (4, 194). However, elevated acetate levels have been consistently associated with obesity due to their role in activating the parasympathetic nervous system, which stimulates hyperphagia and metabolic dysregulation (4, 195). Another study (196) demonstrated that increased acetate turnover in response to a high-fat diet leads to excessive ghrelin and insulin secretion, exacerbating lipid accumulation, hypertriglyceridemia, and systemic insulin resistance. Notably, maternal metabolic disturbances, including obesity and gestational diabetes, are established risk factors for PPD, possibly due to the interplay between metabolic dysfunction, gut microbial alterations, and neuroinflammation (4, 195, 196) (Figures 2, 3).

### Epigenetic regulation mediated by SCFAs, diet, and postpartum mood disorders

SCFAs also exert profound effects on epigenetic programming. A comprehensive analysis of 55 unique and combinatorial histone post-translational modifications (PTMs) revealed that gut microbial colonization modulates both histone acetylation and methylation patterns (4, 197). Notably, these epigenetic changes were tissue-specific and influenced by dietary composition. Mice fed a high-fat, high-sugar diet exhibited reduced histone PTM levels compared to those on a fiber-rich diet, suggesting that diet-induced alterations in microbial-derived SCFAs can influence host epigenetic landscapes. Furthermore, SCFA supplementation in germ-free mice partially replicated the histone modification patterns observed in conventionally colonized mice (4, 197).

In the context of postpartum depression, SCFA-mediated epigenetic modifications may influence maternal stress resilience and neurotransmitter regulation. Butyrate, a HDAC inhibitor, has been shown to exert antidepressant-like effects by promoting histone acetylation and increasing BDNF expression (4, 197). Given that BDNF plays a crucial role in neuroplasticity and mood regulation, disruptions in SCFA production due to maternal gut dysbiosis could contribute to PPD vulnerability. However, whether these epigenetic changes are transgenerationally inherited or established in utero remains an open question. Future research exploring the link between maternal diet, gut microbial SCFAs, and postpartum mental health may provide novel dietary interventions to mitigate PPD risk (4) (Figures 2, 3). Parallel evidence from MDD and stress-related psychiatric disorders highlights the contribution of epigenetic mechanisms to mood regulation and stress responsivity (86–88). These studies consistently demonstrate that histone post-translational modifications (PTMs) and DNA methylation changes are sensitive to environmental inputs, including stress and metabolic factors. Importantly, several investigations have shown that such epigenetic modifications are tissue-specific and strongly influenced by dietary composition. For example, rodents exposed to high-fat, high-sugar diets exhibit reduced histone PTM levels compared with those maintained on fiber-rich diets, implicating microbial-derived short-chain fatty acids (SCFAs) as key mediators of host epigenetic regulation (4, 197). SCFA supplementation in germ-free mice partially recapitulates the histone modification profiles observed in conventionally colonized animals, further supporting a causal role for the gut microbiota.

In the context of postpartum depression, SCFA-mediated epigenetic modulation represents a biologically plausible, yet underexplored, pathway linking diet, gut microbiota, and maternal mental health. Butyrate, a well-characterized HDAC inhibitor, has demonstrated antidepressant-like effects in preclinical models through enhanced histone acetylation and increased BDNF expression. While these findings derive largely from non-postpartum models, they offer a mechanistic framework for investigating how microbiota-driven epigenetic regulation may influence maternal stress resilience and neurotransmitter systems during the postpartum period. Targeted studies in postpartum populations are needed to establish the specificity and translational relevance of these mechanisms to PPD.

### Specific microbiome and epigenetic-based therapeutic strategies for PPD

PPD is characterized by complex interactions among neuroendocrine, immune, microbial, and epigenetic factors. While broad claims about microbiome and epigenetic therapies are promising, recent high-quality evidence now allows for more precise recommendations across four domains: probiotics/postbiotics, fecal microbiota transplantation, epigenetic modulators/dietary methyl donors, and tailored nutrition strategies.

*Probiotics and postbiotics tested in PPD*: Emerging clinical and preclinical data support the feasibility of probiotic supplementation as an adjunctive strategy to mitigate depressive symptoms in postpartum women. Probiotics such as live beneficial bacteria can modulate systemic inflammation, influence neuroactive metabolite production (e.g., short-chain fatty acids), and improve stress-related behavioral outcomes. (1) In a randomized controlled trial, perinatal probiotic administration (*Lactobacillus rhamnosus* HN001) significantly reduced postpartum depressive and anxiety scores compared with controls, suggesting beneficial modulation of the gut-brain axis in this population (60). (2) Mechanistic studies reveal that supplementation with *Lactobacillus* and *Bifidobacterium* strains increases circulating levels of anti-inflammatory metabolites and enhances hippocampal expression of brain-derived neurotrophic factor (BDNF), a key neuroplasticity mediator often suppressed in PPD (198).

*Postbiotics*: non-viable bacterial products such as SCFAs or heat-killed probiotic derivatives also show promise against neuropsychiatric disorders. Butyrate and other SCFAs exert anti-inflammatory and epigenetic regulatory effects through inhibition of histone deacetylases, offering therapeutic potential for restoring neural plasticity and reducing perinatal inflammation (199, 200). Together, these findings suggest that targeted probiotic or postbiotic formulations could serve as safe, non-pharmacological adjuncts to conventional PPD therapies, with ongoing trials now evaluating strain-specific and dose-dependent effects.

*Fecal microbiota transplantation (FMT)*: the transfer of microbial communities from healthy donors to recipients has revolutionized treatment for recurrent *Clostridioides difficile* infection and is now being explored for neuropsychiatric disorders (167). While direct clinical evidence for FMT in PPD remains limited, several compelling preclinical studies support its potential. In mouse models of stress-induced depressive behavior, FMT from healthy donors normalized behavioral deficits, reduced neuroinflammation, and restored gut barrier integrity compared with FMT from depressed donors (9, 201). Similarly, FMT ameliorated behavioral and immune abnormalities in rodent models of *maternal microbiota dysbiosis*, indicating that reconstitution of microbial ecosystems can directly influence central inflammatory pathways implicated in PPD (9, 167, 201). Although human FMT trials in PPD are not yet published, these mechanistic data provide a rationale for exploring microbiota reconfiguration as a novel intervention in at-risk postpartum populations, particularly when standard treatments are ineffective or contraindicated.

*Epigenetic drugs and dietary methyl donors*: Epigenetic therapies modulate chromatin states, DNA methylation, and gene expression. In mood disorders, several candidate strategies have emerged as pharmacological epigenetic modulators. HDAC inhibitors, including sodium butyrate, valproic acid, and newer small molecules, increase histone acetylation at plasticity genes such as BDNF, enhancing resilience to stress and depressive behaviors in preclinical models. Butyrate is particularly relevant as it overlaps with microbial metabolite pathways. More selective class I HDAC inhibitors are under investigation for their antidepressant effects with improved safety profiles compared with broad agents (202, 203).

*Dietary methyl donors*: (1) Folate, choline, and betaine serve as essential cofactors in one-carbon metabolism and S-adenosylmethionine (SAM) synthesis—the universal methyl donor for DNA and histone methylation. Adequate maternal intake of these nutrients influences methylation at key stress regulatory loci such as NR3C1 and FKBP5 and is linked to reduced depression risk (85, 204, 205). Small trials suggest that perinatal methyl donor supplementation may lower inflammatory markers and support mood stability, although larger mechanistic studies in PPD populations are needed. These interventions represent a translational bridge between microbial metabolites, epigenetic control, and mood regulation, and emphasise the potential of combined microbial-epigenetic therapeutic strategies (85, 204, 205).

*Precision nutrition strategies for postpartum women*: Nutrition exerts profound influence on the gut microbiome, systemic inflammation, and epigenetic regulation. Precision nutrition is the tailoring of dietary recommendations based on individual microbial, metabolic, and genetic profiles which holds promise for PPD prevention and management. (1) Diets rich in prebiotic fiber, omega-3 fatty acids, and polyphenols are associated with increased abundance of beneficial microbiota, improved SCFA production, and reduced systemic inflammation factors that correlate with lower depressive symptoms in perinatal cohorts (8). (2) Interventions incorporating Mediterranean-style diets, high in fiber and anti-inflammatory components, improve mood and cognitive outcomes in postpartum women, with emerging evidence for modulation of both microbial and epigenetic pathways.

Personalized dietary plans based on gut microbiome profiling can optimize SCFA production, ensure sufficient methyl donor intake, and target metabolic pathways implicated in PPD, effectively integrating both microbiome and epigenetic mechanisms (8, 183–185). In contrast to a general therapeutic overview, current evidence now supports specific microbiome-based, epigenetic, nutritional, and precision medicine interventions with mechanistic links to PPD biology. While many of these strategies require further clinical validation, the convergence of high-quality preclinical and early clinical data establishes a clear roadmap for next-generation PPD therapies that target microbial ecosystems and chromatin regulation simultaneously (8, 183–185).

## Conclusion

Postpartum depression is a multifactorial disorder influenced by genetic, epigenetic, hormonal, immune, and environmental factors. While traditional research has primarily focused on hormonal and psychosocial contributors, emerging evidence underscores the significant role of gut microbiota and the microbiota-gut-brain axis in modulating maternal mental health. This review highlights the intricate interplay between genetics, epigenetics, and gut microbiota, revealing their collective impact on neuroimmune signaling, neurotransmitter synthesis, and epigenetic regulation.

Key findings suggest that alterations in gut microbiota composition during the peripartum period may disrupt neuroimmune homeostasis, contributing to increased inflammation, oxidative stress, and dysregulated neurotransmitter pathways, all of which have been implicated in PPD. Additionally, epigenetic modifications, including DNA methylation and histone alterations, further mediate gene expression changes that influence PPD susceptibility. Understanding these molecular mechanisms offers novel insights into PPD pathophysiology, paving the way for targeted therapeutic strategies.

Furthermore, the role of choline metabolism in epigenetic regulation presents a compelling avenue for exploring fetal programming and transgenerational effects on maternal and infant mental health. These findings suggest that gut microbiota modulation, through dietary interventions, probiotics, and prebiotics, may serve as a promising strategy for mitigating PPD risk and improving maternal mental well-being.

### Future directions

Despite significant progress in understanding the biological mechanisms underlying PPD, several knowledge gaps remain. Future research should address the following key areas: (1) Multi-omics integration in PPD research: The integration of genomics, epigenomics, transcriptomics, proteomics, and metabolomics is essential to comprehensively understand the complex molecular landscape of PPD. Advanced computational models and artificial intelligence (AI)-driven approaches can help identify predictive biomarkers for early diagnosis and intervention. (2) Longitudinal studies on gut microbiota and PPD progression: Large-scale, longitudinal cohort studies are needed to explore dynamic changes in gut microbiota composition from pregnancy to postpartum and their correlation with mental health outcomes. Investigating the resilience and adaptability of the gut microbiome in response to external factors such as diet, stress, and environmental exposures may provide insights into personalized intervention strategies. (3) Role of microbiota-derived metabolites in PPD pathophysiology: Short-chain fatty acids (SCFAs), tryptophan metabolites, and gut-derived neurotransmitters (e.g., serotonin, GABA) warrant further exploration to determine their specific contributions to maternal mood regulation. Identifying microbial species responsible for the production of neuroactive metabolites could enable the development of targeted probiotic therapies. (4) Epigenetic mechanisms in gut-mediated PPD pathways: Future studies should investigate how gut microbiota modulate DNA methylation, histone modifications, and non-coding RNA expression in genes associated with neuroinflammation and depression. The transgenerational effects of maternal gut microbiota on offspring neurodevelopment and mental health should be explored through epigenetic imprinting mechanisms. (5) Gut microbiota-based therapeutic approaches: Probiotic, prebiotic, and postbiotic interventions tailored to modulate maternal gut microbiota composition should be evaluated in clinical trials for their efficacy in preventing or alleviating PPD symptoms. Investigating fecal microbiota transplantation (FMT) as a potential intervention for severe cases of PPD may provide novel treatment avenues. (6) Influence of choline metabolism on maternal and infant health: The role of dietary choline in modulating maternal gut microbiota, epigenetic modifications, and neurodevelopmental outcomes in offspring should be further explored. Controlled dietary supplementation studies should assess whether optimizing choline intake during pregnancy and postpartum can improve mental health outcomes. (7) Personalized medicine and precision psychiatry in PPD management: Identifying genetic and microbiome-based risk profiles for PPD could enable the development of personalized therapeutic interventions. Pharmacogenomics and microbiome-driven therapeutic approaches should be explored to optimize antidepressant efficacy and minimize adverse effects in postpartum women.

The intersection of genetics, epigenetics, and the microbiota-gut-brain axis represents an exciting frontier in postpartum depression research. By bridging the gap between molecular psychiatry and microbiome science, future studies have the potential to revolutionize PPD prevention, diagnosis, and treatment. Leveraging multi-omics approaches, personalized medicine, and gut-targeted therapeutics could lead to novel, effective strategies for mitigating PPD and enhancing maternal–infant well-being. A deeper understanding of these biological underpinnings will not only refine clinical interventions but also foster a more holistic approach to maternal mental health care.

## Data Availability

The original contributions presented in the study are included in the article/supplementary material, further inquiries can be directed to the corresponding author.
